# Improvement in motor symptoms, physical fatigue, and self-rated change perception in functional motor disorders: a prospective cohort study of a 12-week telemedicine program

**DOI:** 10.1007/s00415-022-11230-8

**Published:** 2022-07-09

**Authors:** Marialuisa Gandolfi, Angela Sandri, Christian Geroin, Federica Bombieri, Marianna Riello, Zoe Menaspà, Chiara Bonetto, Nicola Smania, Michele Tinazzi

**Affiliations:** 1 Department of Neurosciences, Biomedicine and Movement Sciences, University of Verona, Verona, Italy; 2 Neuromotor and Cognitive Rehabilitation Research Centre (CRRNC), University of Verona, Verona, Italy; 3 Department of Neurosciences, Biomedicine and Movement Sciences, Neurology Unit, University of Verona, Verona, Italy; 4 Department of Neurosciences, Biomedicine and Movement Sciences, Section of Psychiatry, University of Verona, Verona, Italy

**Keywords:** Telemedicine, Motor symptoms, Physical fatigue, Quality of life, Gait disorders, Depression, Anxiety

## Abstract

**Background:**

Functional motor disorders (FMDs) are highly disabling conditions associated with long-term disability, poor quality of life, and economic burden on health and social care. While multidisciplinary 5-days rehabilitation programs have been shown to reduce motor and non-motor symptoms, long-term management and monitoring in FMDs remain an unmet need.

**Aim:**

To compare a 12-weeks telemedicine program against a 12-weeks self-management program after a 5-days rehabilitation program for improving motor, non-motor symptoms, quality of life, and perception of change in patients with FMDs.

**Methods:**

The study population was 64 consecutive patients with a definite diagnosis of FMDs who underwent a 5-days in-person rehabilitation program followed by either a self-management (the first 32 patients) or a telemedicine program (the latter 32 patients). Validated measures of motor and non-motor symptoms such as fatigue and pain, quality of life, perception of change, gait, and postural control were recorded before (T0), after completion of rehabilitation (T1), and then again at 3 months (T2).

**Results:**

Improvement at 3-month follow-up assessment of motor symptoms (*p* < 0.001), physical fatigue (*p* = 0.028), and self-rated change perception (*p* = 0.043) was greater in the telemedicine group. No different between-groups effect was found on other dimensions of fatigue, pain, physical and mental health, and gait and postural control.

**Conclusions:**

Long-term management and expert monitoring of patients with FMDs via telemedicine may enhance long-term outcomes in motor symptoms and physical fatigue, with a positive long-term impact on self-rated health perception of change.

## Introduction

Functional motor disorders (FMDs) are considered within a wide category of functional neurological disorders (FNDs). They are characterized by abnormal movement (gait, dystonia, weakness, balance disorders, tremor) that can be altered by distraction or non-physiological maneuvers; they are clinically incongruent with organic neurological disease movement disorders [1, 2]. The incidence ranges from 4 to 12 per 100,000 population per year, with a high prevalence (15–20%) in patients attending a neurological clinic [2–5]. These highly disabling conditions are associated with long-term disability, poor quality of life and distress just like patients with movement disorders, and economic burden on health and social care [6–11]. Beside motor complaints and abnormal movement, non-motor symptoms (NMSs, pain, fatigue, anxiety, depression, alexithymia, cognitive complaints) greatly contribute to disability and distress, reducing quality of life (QoL) and affecting treatment outcomes [7, 12–16]. Widely misunderstood, FMDs have received little general attention and cross-country clinical research into their management has been inconsistent [17–20].

FMDs pathophysiology and management have not yet been elucidated [3, 4, 21]. Attention, sense of agency, and belief/expectations are implicated in FMD pathogenesis: abnormal movement’s prediction is triggered by self-focused attention and the resulting movement is generated without the implicit normal sense of agency [21–24]. Moreover, psychological and neurobiological factors have been integrated into a biopsychosocial framework of predisposing factors, neural networks, and environmental influence [25]. Within this perspective, the goal of rehabilitation is to reduce disability and improve health-related quality of life [HR-QoL, Healthcare Improvement Scotland. Stepped care for functional neurological symptoms. Heal Improv Scotl. 2012; (February 2022)] [13, 26, 27]. Multidisciplinary rehabilitation is essential to improve function and QoL in patients with FMDs [13]. Such patients may have much greater potential for recovery than health-care workers often think possible [13, 28]. For example, based on the biopsychosocial model, intensive 5-day programs in FMDs have proved efficacious in improving motor and non-motor symptoms, with follow-up at 3 and/or 6 months [16, 24, 29, 30]. The programs have been accepted as an essential tool in FMDs management [16, 30–32]; nonetheless, long-term management and monitoring needs remain unmet [16, 19]. Patients generally feel misunderstood and neglected by health-care professionals and risk becoming progressively more vulnerable [33]. We recently reported that an intensive 5-days rehabilitation program followed by a tailored, home-based self-management plan failed to stabilize a reduction in NMSs as measured at the 3-months follow-up assessment: the patients reported greater general and physical fatigue and no relief in depressive and anxiety symptoms or mental health [24].

Given the impact of NMSs on HR-QoL [15], providing further tailored support is essential while patients integrate the knowledge they acquired on a 5-days rehabilitation program into their daily routine. Demartini et al. [34] reported that physiotherapy combined with telemedicine may have a valuable role in improving motor symptom severity, self-assessment of outcome (as measured on the Clinical Global Impression scale), and HR-QoL in patients with FMDs. The study’s main limitations were the lack of evidence-based intensive rehabilitation, the small sample size, and the lack of a control group. Nevertheless, they found that a telemedicine program is feasible and safe also in FMDs patients [34, 35] and that it can overcome distance and time barriers, thus providing access to patients with temporary or permanent disabilities for their accurate monitoring by rehabilitation experts prescribing and delivering rehabilitation [36].

With the present study we compared the effect of a 12-weeks telemedicine program against a 12-weeks self-management program (control group) after completion of a multidisciplinary 5-days rehabilitation program for motor and non-motor symptoms, quality of life, and perception of change in patients with FMDs.

## Materials and methods

### Study design

**Fig. 1:**
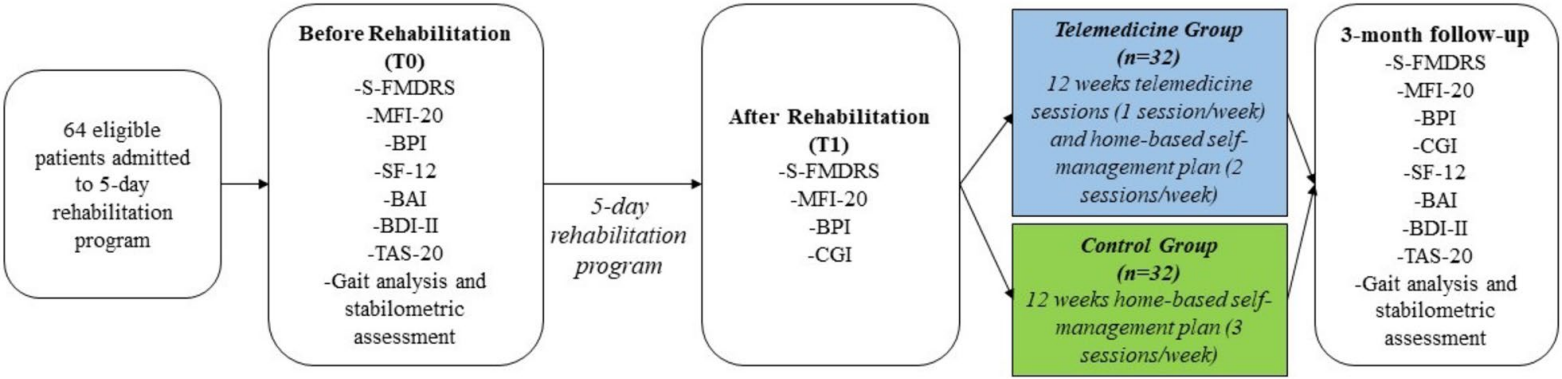
Study design and measures. *S-FMDRS* Simplified Functional Movement Disorders Rating Scale, *MFI-20* Multidimensional Fatigue Inventory scale, *BPI* Brief Pain Inventory, *SF-12* 12-Item Short-Form Health survey, *BAI* Beck Anxiety Inventory, *BDI-II* Beck Depression Inventory-II, *TAS-20* Toronto Alexithymia Scale, *CGI* Clinical Global Impression scale

For this prospective cohort study, we included consecutive patients with a clinically established diagnosis of FMDs [37] attending the Parkinson’s Disease and Movement Disorders Unit (AOUI Verona, Italy) between June 2019 and December 2021. All patients underwent a 5-days in-person rehabilitation program. They were assessed before rehabilitation (T0), at the completion of the program (T1), and then at 3-months follow-up assessment (T2). Between T1 and T2, the telemedicine group attended a 12-weeks tele-session program (1 session/week), while the control group followed a home-based self-management program (Fig. 1, Table 1). We performed this explorative feasibility study before implementing the randomized controlled trial [NCT05345340].

**Table 1:** Rehabilitation and home-based self-management program

The 5-day in-person rehabilitation program		
Treatment principles	Treatment explanation	Expected impact
Education	Give information about diagnosis and explain symptoms according to the pathophysiological model of FMDs	To reinforce information about diagnosis for better comprehension of symptoms according to the pathophysiological model of FMDs
Exploration of how symptoms affect movement and posture	Explore how symptoms affect movement and posture and demonstrate how positive signs (e.g., Hoover’s sign for weakness or entrainment for tremor) can aid patients in developing strategies to overcome abnormal movement patterns	To engage patients in diagnosis and treatment to regain normal movement while learning to manage their symptoms
Retraining movement using strategies based on redirection of attention	Cognitive (counting or arithmetic) or physical (finger tapping or hand prono-supination) exercises during concurrent meaningful tasks (i.e., walking)	To reduce self-focused attention and increase external-focused attention
	Graded exercises, visualization techniques with mirrors and video observation, and distraction maneuvers during meaningful tasks (e.g., sit-to-stand, transfers, forward and backward walking or sideways, weight bearing while progressively reducing upper limb support, upper and lower limb coordination exercises in static (quiet stance) and dynamic (during gait) conditions using a ball, treadmill walking and with visual feedback using a mirror Walking aids, splints, and orthoses preferably avoided to prevent interference from adaptive behaviors	
Development of a self-management plan	Exercises tailored to the patient’s needs and condition performed during the 5-day rehabilitation program are reported on a paper log and video recorded It includes goals, activity plans, and strategies to retrain movements and redirect attention. Videos include exercise demonstration and execution and strategies to retrain movements	To facilitate acquisition of the program’s educational components and promote patient engagement in treatment goals
Home-based self-management plan		
Type of symptoms	Task explanation	Expected Impact
Lower limb weakness	Overground walking at different gait speeds with/without obstacles, walking backward and sideways. Sit-to-stand activities and squat exercises	Improve lower limb function in daily life activities
Gait and balance symptoms	Overground walking at different gait speeds with/without obstacles, backward and side walking. Increase and decrease walking speed. Side-to-side weight shift. Walking carrying objects, catching and throwing a ball, walking with high steps. In stance, over compliant surfaces, according to patient’s abilities	Improve mobility and endurance
Upper limb weakness	Bear weight on the hands (i.e., 4-point kneeling or standing with hands on a table/surface). Improve use of the weaker upper limb in daily life activities (i.e., use of mobile, tablet, cutlery) Straddling a Swiss ball, with feet placed shoulder width apart and keeping balance to stimulate automatic upper limb postural responses	Improve upper limb function and use in daily life activities
Dual-task activities	Keep walking, catching, and throwing a ball Keep walking quickly, changing direction (forward, backward, sideways). Keep walking, bouncing a ball alternatively with right and left hand. Keep walking, while increasing leg movement amplitude (greater stride length) and swing movement of arms. Keep walking and kayaking movements using a stick	Improve correct use of cognitive and motor strategies during static and dynamic conditions to reduce self-focused attention and increase external-focused attention
Tremor and dystonia	Making the movement voluntary by tremoring, increase movement amplitude while reducing frequency, then gradually slowing the movement to a stop, teaching patients how to relax their muscles by actively contracting them for a few seconds, then relaxing in front of a mirror. Focus on another body part, for example, tapping the other hand or foot. Teach strategies to turn overactive muscles off while sitting or lying	Reduce symptom severity and occurrence

### Participants

All patients were referred by the same consultant neurologist who had made the diagnosis (MT). Inclusion criteria were: established diagnosis of FMDs [37], age ≥ 18 years, completion of the 5-days rehabilitation program, acceptance of diagnosis, and access to a computer or a smartphone with Internet connection. Exclusion criteria were: prominent dissociative seizures, prominent cognitive and/or physical impairment that precluded signing the informed consent form for study participation based on clinical judgment, having discontinued the 5-days rehabilitation program or the telemedicine program (for the telemedicine group), and incomplete assessment and questionnaire because of language comprehension difficulties. The convenience sample consisted of consecutive patients enrolled between June 2019 and June 2020 for the self-management group and after July 2020 for the telemedicine group.

### Intervention

All patients attended the in-person 5-days rehabilitation program (2 h/day) to re-establish normal movement patterns within a multidisciplinary etiological framework according to a validated rehabilitation protocol for FMDs [24, 30, 31]. Details on the intervention have been described previously and are reported in Table 1 [24]. Rehabilitation was delivered by the same physiotherapist trained in FMDs treatment (CG). On completion of the 5-days in-person rehabilitation program, the patients were enrolled in either the telemedicine program or the home-based self-management plan [24, 31].

### Self-management

The patients underwent a 5-days in-person rehabilitation program followed by a home-based self-management plan according to clinical practice [30, 31]. The aim of the home-based self-management plan was to facilitate acquisition of the program’s educational components and promote a self-management approach to treatment after discharge. The plan was implemented during the 5-days rehabilitation with exercises deemed helpful for the patients and included a paper log and video. The self-management plan was explained and discussed with all patients and their caregivers at the end of the 5-day in-person rehabilitation program (Table 1). The patients were encouraged to perform the self-management program at home (1 h/day, 3 days/week) for 12 consecutive weeks on their own or with the help of their caregivers.

### Telemedicine intervention

In July 2020, the multidisciplinary team introduced a telemedicine protocol for FMDs at the Parkinson’s Disease and Movement Disorders Unit at the end of the 5-days program [34]. The control group patients were instructed to perform three home-based sessions of exercises per week (1 h/day per session). The telemedicine intervention was delivered by the same therapist trained in FMDs treatment (FB) during one out of the three weekly home-based sessions for 12 consecutive weeks (30 min/session, 1 session/week). During each session, the therapist had a video call with the patient via a smartphone or a computer connection according to the patient’s availability and preference. The therapist monitored the patient going through the exercises, giving feedback on execution, with further instructions at the end of the session regarding changes in exercise type/intensity/frequency according to the patient’s improvement and feedback. The patients were asked to video record their performance of challenging exercises during the week to document any difficulties or adverse events to be discussed with the therapist during the next tele-session.

### Outcome measures

Patient demographics (age, sex) and detailed clinical history (i.e., motor and NMSs onset, disease duration, neurological, psychiatric or medical comorbidities, previous organic diagnosis) were collected at admission according to the Italian Registry for FMDs [7].

### Motor and NMSs outcomes

Motor symptom severity and duration were measured with the objective-rated Simplified Functional Movement Disorders Rating Scale (S‐FMDRS; range 0–54; higher score means more motor symptoms) [30]. Fatigue was assessed with the Multidimensional Fatigue Inventory Scale (MFI-20) [38], differentiating between general and physical fatigue, reduced motivation and activity, and mental fatigue (subscales range 4–20; higher score means more perceived fatigue). We assessed pain and neuropsychological measures with self-rated scales: Brief Pain Inventory (BPI, Intensity subscale range 0–40, higher score means worse pain intensity; Interference subscale range 0–70, higher score means worse pain interference in daily activities) [39]; Beck Depression Inventory (BDI-II, range 0–63, higher score means more depressive symptoms) [40]; Beck Anxiety Inventory (BAI, range 0–63, higher score means more anxiety symptoms) [41]; and Toronto Alexithymia Scale (TAS-20, range 20–100, higher score means more difficulties in identify, recognize and express emotions) [42]. Patients with gait and balance disorders underwent instrumental assessment using the GaitRite walkway system (CIR Systems Inc, Havertown, PA, USA) and an electronic monoaxial stabilometric platform (Technobody ©, Dalmine, Italy). Measures of gait were gait speed (cm/s), cadence (step/min), and stride length (cm). Measures of postural control were length of the center of pressure (CoP) trajectory (mm) and sway area (mm^2^) measured in eyes open (EO) and in eyes closed (EC) condition. The EO condition integrates visual, proprioceptive, and vestibular contributions to postural stability, whereas the EC condition refers to proprioceptive contribution and visual dependency on postural control. Each condition lasted 30 s.

### Quality of life outcomes

QoL was assessed with the self-rated Mental Health and Physical Functioning scale of the 12-item Short-Form Health Survey (SF-12, range 0–100, higher score means better mental and physical health) [43]. Self-rated perception of change after treatment was assessed with the 7-point Clinical Global Impression (CGI) scale; score range from 1 (very much improved) to 7 (very much worse) [44].

Assessment with S‐FMDRS, BPI, and MFI-20 was performed at T0, T1, and T2. Neuropsychological outcomes (BAI, BDI-II, TAS), SF-12, and gait and balance were evaluated at T0 and T2. CGI was recorded at T1 and T2. A flowchart of the interventions and data collection at the three time points are reported in Fig. 1.

### Statistical analysis

Descriptive statistics included frequencies for categorical variables and means and standard deviations for continuous variables. Comparisons between groups were performed by Chi-square or Fisher’s exact test for categorical variables and independent t test for continuous variables. Two-way mixed ANOVA was used to analyze the main factor “Time” as a within-subject factor (T0, T1, T2) and “Group” (telemedicine and control group) as a between-group factor. Post hoc analyses were performed using a *t* test and applying Bonferroni correction. All tests were bilateral at *p* < 0.05. Analyses were performed with RStudio software (Version 1.3.1093 © 2009–2020 Rstudio, PBC).

## Results

The demographical and clinical features of the 64 patients are reported in Table 2. There were no differences in age, sex, disease duration, motor and NMSs presentation (all, *p* > 0.05), except for a greater number of gait impairment symptoms (*p* = 0.009) and combined phenotypes (*p* = 0.003) in the telemedicine group. All patients completed assessment at all three time points, except for gait and balance assessment data; data were incomplete or missing for 21 patients because of the COVID-19 pandemic or technical problems. The self-management group was consecutively enrolled from June 2019 to July 2020, while the telemedicine group was consecutively enrolled between July 2020 and December 2021.

**Table 2:** Main demographic and clinical characteristics of the study sample before the 5-day in-person rehabilitation program (T0) (*n* = 64)

	All patients (*n* = 64)	Telemedicine (*n* = 32)	Control (*n* = 32)	Between-group analyses, *p*
Mean age, years (± SD)^a^	40.77 (14.61)	38.84 (12.76)	42.70 (16.22)	0.29
Women, no. (%)^b^	54 (84)	30 (94)	24 (75)	0.08
Mean duration symptoms, years (± SD)^a^	3.75 (3.88)	3.46 (3.48)	4.04 (4.28)	0.56
***Clinical characteristics–no. (%)***				
*Motor symptoms*^b^				
Tremor	37 (58)	22 (69)	15 (47)	0.13
Weakness	52 (81)	27 (84)	25 (78)	0.75
Dystonia	17 (27)	9 (28)	8 (25)	1
Myoclonus	7 (11)	3 (9)	4 (12)	1
Facial disorders	13 (20)	10 (31)	3 (9)	0.06
Gait impairments	41 (64)	26 (81)	15 (47)	0.009*
Voice disorders	15 (23)	9 (28)	6 (19)	0.56
Swallowing disorders	7 (11)	6 (19)	1 (3)	0.10
*FMDs phenotype*^b^				
Isolated	12 (19)	1 (3)	11 (3)	0.003*
Combined	52 (81)	31 (97)	21 (66)	
NMSs^b^				
Reported fatigue	47 (73)	24 (75)	23 (72)	1
Reported chronic pain	44 (69)	26 (81)	18 (56)	0.06
***Previous organic disease/comorbidities,***^***b§***^				
Neurological disease	20 (31)	11 (34)	9 (28)	0.79
Psychiatric disease	9 (14)	3 (9)	6 (19)	0.47
Medical disease	34 (53)	16 (50)	18 (56)	0.80

*no.* number, *SD* standard deviation, *NMSs* non-motor symptoms, *FMDs* functional motor disorders, *p p*-value

^a^For statistical tests such as Two Sample independent *t*-test

^b^Chi-squared test, or Fisher’s Exact test

^§^Patients can have one or more organic disease/comorbidities

^*^p < 0.05

### Motor outcomes

**Fig. 2:**
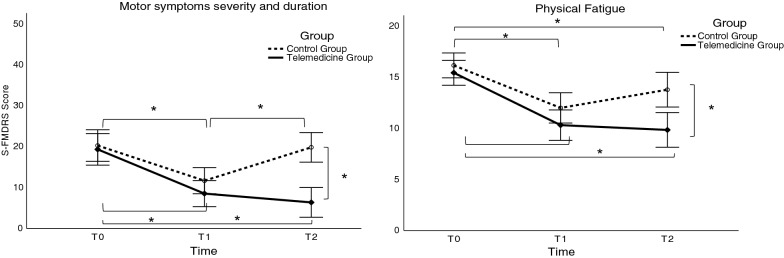
Motor and NMSs symptom severity in the telemedicine and the control group before, at completion of the 5-days in-person rehabilitation program, and at the 3-months follow-up. T0 before initiating the 5-days in-person rehabilitation; T1 at completion of the 5-days in-person rehabilitation; T2 at the 3-months follow-up. *Statistically significant. *p* value was corrected for multiple comparisons. *S-FMDRS* Simplified Functional Movement Disorders Rating Scale; Physical Fatigue, a subscale of the MFI-20 (Multidimensional Fatigue Inventory-20)

The analysis yielded a significant Time × Group interaction for motor symptoms severity on the S-FMDRS scale [*F* (2, 124) = 18.608, *p* < 0.001] (Table 3, Fig. 2). Post hoc comparison revealed a reduction in motor symptom severity scores (improvement) in both groups at T1 compared to T0 (all *p* < 0.001), but only the telemedicine group maintained improvement at T2 compared to T0 (mean difference 12.97 ± 1.63; *p* < 0.001). Between-group post hoc comparison revealed significantly lower motor symptom severity in the telemedicine compared to the control group at T2 (mean difference 13.47 ± 2.56; *p* < 0.001). The effect of Group [*F* (1, 62) = 7.170, *p* = 0.009] and Time [F (2, 124) = 41.073, *p* < 0.001] was also significant (Table 3, Fig. 2). The overall effect of Group revealed significantly lower motor symptom severity in the telemedicine (total mean over time 11.38 ± 1.55) compared to the control group (total mean over time 17.24 ± 1.55). The overall time effect revealed a decrease in overall motor symptom severity at T1 and T2 irrespective of Group by 9.73 (± 1.02) and 6.7 (± 1.15) points compared to T0, respectively (all *p* < 0.001). No other comparisons were statistically significant (all *p* > 0.52). Analysis of spatio-temporal gait and stabilometric parameters showed no significant effect of group or interaction (all *p* > 0.081). There was a main effect of time for gait speed [*F* (1, 42) = 30.273, *p* < 0.001], cadence [*F* (1, 42) = 7.915, *p* = 0.007], stride length [*F* (1, 42) = 32.661, *p* < 0.001], CoP displacement in the eyes open [*F* (1, 41) = 6.133, *p* = 0.017 and eyes closed [*F* (1, 41) = 8.120, *p* = 0.007 condition. Performance was better at T2 than at T0 (all *p* < 0.019) for both groups (Table 4).

**Table 3:** Motor and NMSs outcome measures before (T0), after (T1) the 5-day in-person rehabilitation program, and at the 3-month follow-up (T2) (*n* = 64)

Outcomes	Before—T0		After—T1		Follow-up—T2		Intervention phase			Repeated-measures mixed ANOVA		
	Mean (SD)		Mean (SD)		Mean (SD)		Between-group difference (95% CI) Mean (LB, UB)			Group	Time	Time × Group
	Telemedicine	Control	Telemedicine	Control	Telemedicine	Control	Before	After	Follow-up	*p*	*p*	*p*
S-FMDRS (0–54)	19.31 (10.62)	20.25 (11.20)	8.5 (9.27)	11.66 (8.83)	6.34 (7.28)	19.81 (12.55)	− 0.94 (− 5.67, 3.79)	− 3.16 (− 7.42, 1.10)	− 13.47 (− 18.03, − 8.91)	0.009*	< 0.001*	< 0.001*
*MFI-20*												
General fatigue (4–20)	15.13 (3.45)	16.22 (3.14)	10.53 (3.94)	12.09 (4.45)	10.75 (4.30)	14.34 (5.11)	− 1.09 (− 3.91, 1.73)	− 1.56 (− 4.61, 1.49)	− 3.59 (− 6.77, − 0.41)	0.01*	< 0.001*	n.s
Physical fatigue (4–20)	15.44 (3.49)	16.16 (3.41)	10.31 (3.77)	12.00 (4.60)	9.84 (4.91)	13.78 (4.70)	− 0.72 (− 3.58, 2.14)	− 1.69 (− 4.74, 1.36)	− 3.94 (− 7.14, − 0.74)	0.01*	< 0.001*	0.023*
Reduced activity (4–20)	13.19 (3.86)	14.22 (4.22)	9.22 (3.67)	11.78 (4.61)	9.13 (4.38)	11.63 (4.86)	− 1.03 (− 4.04, 1.98)	− 2.56 (− 5.60, 0.48)	− 2.5 (− 5.65, 0.65)	0.016*	< 0.001*	n.s
Reduced motivation (4–20)	9.13 (3.81)	10.25 (3.72)	6.97 (2.89)	9.31 (3.32)	7.44 (3.85)	10.00 (3.89)	− 1.12 (− 4.06, 1.82)	− 2.34 (− 5.12, 0.44)	− 2.56 (− 5.53, 0.41)	0.006*	0.007*	n.s
Mental fatigue (4–20)	12.19 (5.06)	12.50 (3.76)	10.19 (3.93)	11.19 (3.63)	10.28 (4.51)	11.91 (4.35)	− 0.31 (− 3.42, 2.80)	− 1 (− 3.94, 1.94)	− 1.63 (− 4.74, 1.48)	n.s	0.003*	n.s
*BPI*												
Intensity (0–40)	20.69 (14.80)	17.91 (12.04)	15.88 (12.54)	17.56 (11.68)	15.44 (13.30)	18.63 (13.11)	2.78 (− 2.59, 8.15)	− 1.68 (− 6.71, 3.35)	− 3.19 (− 8.49, 2.11)	n.s	n.s	n.s
Interference (0–70)	31.13 (24.21)	27.47 (24.82)	22.34 (22.33)	24.22 (23.64)	20.84 (20.76)	27.88 (24.57)	3.66 (− 4.47, 11.79)	− 1.88 (− 9.63, 5.87)	− 7.04 (− 14.72, 0.64)	n.s	n.s	n.s

*S-FMDRS* Simplified Functional Movement Disorders Rating Scale, *MFI-20* Multidimensional Fatigue Inventory-20, *BPI* Brief Pain Inventory, *SD* standard deviation, *CI* confidence interval, *LB* lower bound, *UB* upper bound

*Statistically significant. *p*-value was adjusted for multiple comparisons; n.s., not significant

**Table 4:** Gait and stabilometric performance before initiating the 5-day in-person rehabilitation program and at the 3-month follow-up

Outcomes	Before—T0		Follow-up—T2		Intervention phase		Repeated measures mixed ANOVA		
	Mean (SD)		Mean (SD)		Between-group difference (95% CI) Mean (LB, UB)		Group	Time	Time × Group
	Telemedicine (*n* = 22)	Control (*n* = 22)	Telemedicine (*n* = 22)	Control (*n* = 22)	Before	Follow-up	*p*	*p*	*p*
*Gait analysis*									
Gait speed (cm/s)	73.56 (32.46)	62.15 (26.51)	92.09 (27.01)	81.02 (23.99)	11.41 (0.46, 22.36)	11.07 (1.35, 20.79)	n.s	< 0.001*	n.s
Cadence (step/min)	89.03 (36.63)	84.25 (20.52)	100.48 (13.30)	95.07 (18.73)	4.78 (-6.19, 15.75)	5.41 (-1.50, 12.32)	n.s	0.011*	n.s
Stride length (cm)	93.42 (23.70)	85.03 (24.42)	108.34 (20.47)	100.55 (19.30)	8.39 (-0.88, 17.66)	7.79 (-0.22, 15.80)	n.s	< 0.001*	n.s

Stabilometric assessment	Telemedicine (*n* = 22)	Control (*n* = 21)	Telemedicine (*n* = 22)	Control (*n* = 21)					
Sway area (mm^2^) eyes open	297.27 (588.67)	305.10 (391.52)	164.64 (271.50)	129.24 (119.51)	− 7.83 (− 163.08, 147.42)	35.4 (− 31.14, 101.94)	n.s	n.s	n.s
Sway area (mm^2^) eyes closed	751.18 (1127.43)	692.62 (724.30)	497.50 (987.10)	584.38 (1640.39)	58.56 (− 234.00, 351.12)	− 86.88 (− 499.53, 325.77)	n.s	n.s	n.s
CoP displacement eyes open	240.64 (177.33)	267.52 (197.04)	200.50 (261.61)	167.62 (88.49)	− 26.88 (− 86.01, 32.25)	32.88 (− 29.29, 95.05)	n.s	0.017*	n.s
CoP displacement eyes closed	414.82 (355.22)	414.81 (269.00)	316.50 (297.96)	291.14 (228.58)	0.01 (− 98.45, 98.47)	25.36 (− 57.93, 108.65)	n.s	0.007*	n.s

*CoP* center of pressure, *SD* standard deviation, *CI* confidence interval, *LB* lower bound, *UB* upper bound

*Statistically significant. *p* value was adjusted for multiple comparisons; n.s., not significant

### NMSs outcomes

There was a significant Time × Group interaction only on the Physical fatigue subscale [*F* (2, 124) = 3.890, *p* = 0.023]. Post hoc comparison revealed a significant decrease in both groups at T1 and T2 compared to T0 (all *p* < 0.026); however, fatigue was significantly lower in the telemedicine group only at T2 (mean difference 3.94 ± 1.2; *p* = 0.002; Table 3, Fig. 2). There was a significant main effect of group as measured on the subscales for MFI-20 General fatigue [*F* (1, 62) = 7.075, *p* = 0.01], Physical fatigue [*F* (1, 62) = 7.101, *p* = 0.01], Reduced activity [*F* (1, 62) = 6.181, *p* = 0.016), and Reduced motivation [*F* (1, 62) = 8.135, *p* = 0.006). Overall, less fatigue was perceived by the telemedicine than the control group (all *p* < 0.016). The main effect of Time was significant as measured on the subscales for MFI-20 General fatigue [*F* (2, 124) = 30.235, *p* < 0.001] and Physical fatigue [F (2, 124) = 35.988, *p* < 0.001], Reduced activity [*F* (2, 124) = 19.725, *p* < 0.001], Reduced motivation [*F* (2, 124) = 5.221, *p* = 0.007], and Mental fatigue [*F* (2, 124) = 6.07, *p* = 0.003). The overall level of fatigue was significantly decreased at T1 and T2 compared to T0 as measured on the subscales for MFI-20 General fatigue, Physical fatigue, and Reduced activity (all *p* < 0.026). There was an overall decrease in fatigue as measured on the MFI Reduced motivation and the Mental subscales only at T1 compared to T0 (all *p* < 0.003). Other comparisons and interactions were not statistically significant (all *p* > 0.05; Table 3). No effect of Group, Time, and Time × Group interaction was found for pain intensity and interference (all *p* > 0.05; Table 3). No significant effects or Time × Group interactions were found for BDI-II, BAI, and TAS-20 outcome measures (all *p* > 0.05; Table 5). A significant effect of Time was found for all psychological scales (BDI-II, BAI, TAS-20), with greater improvement at T2 in both groups (all *p* < 0.001). A significant effect of Group was found only for BDI-II [*F* (1, 62) = 4.636, *p* = 0.035], with lower depression scores for the telemedicine compared to the control group (mean difference for BDI-II 3.55 ± 1.65).

**Table 5:** NMSs and quality of life outcome measures before initiating the 5-days in-person rehabilitation program and at the 3-months follow-up

Outcomes	Before—T0		Follow-up—T2		Intervention phase		Repeated measures mixed ANOVA		
	Mean (SD)		Mean (SD)		Between-group difference (95% CI) Mean (LB, UB)		Group	Time	Time × Group
	Telemedicine	Control	Telemedicine	Control	Before	Follow-up	*p*	*p*	*p*
***Primary outcomes***									
BDI-II (0–63)	11.34 (7.61)	13.09 (7.83)	5.66 (5.22)	11.00 (9.27)	− 1.75 (− 5.68, 2.18)	− 5.34 (− 9.22, − 1.46)	0.035*	< 0.001*	n.s
BAI (0–63)	22.03 (11.66)	21.09 (9.86)	15.09 (10.44)	17.47 (9.93)	0.94 (− 3.76, 5.64)	− 2.38 (− 6.93, 2.17)	n.s	< 0.001*	n.s
TAS-20 (20–100)	54.09 (12.18)	55.84 (11.49)	50.63 (12.84)	53.13 (11.40)	− 1.75 (− 6.71, 3.21)	− 2.5 (− 7.53, 2.53)	n.s	< 0.001*	n.s
***Secondary outcomes***									
*SF-12 (0–120)*									
Physical functioning	27.99 (9.22)	30.45 (9.73)	39.45 (14.34)	36.33 (10.24)	− 2.46 (− 6.83, 1.91)	3.12 (− 1.99, 8.23)	n.s	< 0.001*	n.s
Mental health	43.72 (13.47)	38.67 (11.43)	47.72 (8.54)	39.66 (15.37)	5.05 (− 0.07, 10.17)	8.06 (2.95, 13.17)	0.017*	n.s	n.s

*BDI–II* Beck Depression Inventory, *BAI* Beck Anxiety Inventory, *TAS-20* Toronto Alexithymia Scale, *SF-12* 12-Item Short-Form Health Survey (SF-12), *SD* standard deviation, *CI* confidence interval, *LB* lower bound, *UB* upper bound

^*^Statistically significant. *p* value was corrected for multiple comparisons

### Quality of life outcomes

There was no significant Time × Group interaction for the mental health QoL subscale (*p* > 0.05; Table 5); there was a significant effect of Group [*F* (1, 62) = 6.029, *p* = 0.017) that indicated a significantly better perception of their mental health in the telemedicine group (*p* = 0.005). Analysis yielded a significant effect of Time [*F* (1, 62) = 34.1, *p* < 0.001) on the physical subscale of the SF-12 (Table 5). There was an overall improvement in quality of life at T2 compared to T0 (mean difference 8.66 ± 1.48; *p* < 0.001). Fisher’s exact test revealed no between group differences at T1 (*p* = 0.335) but a significant difference (*p* = 0.043) at T2 in self-perception of change (CGI); such difference suggests a significantly better perception of improvement in the telemedicine group (Table 6).

**Table 6:** Patient-rated perception of change after the 5-days in-person rehabilitation program and at follow-up

CGI change	T1 After			T2 follow-up		
	Telemedicine *N* (%)	Control *N* (%)	Fisher’s exact *p* value	Telemedicine *N* (%)	Control *N* (%)	Fisher’s exact *p* value
Improved	23 (74%)	26 (87%)	0.335	30 (94%)	23 (72%)	0.043*
No change/worse	8 (26%)	4 (13%)		2 (6%)	9 (28%)	

*CGI* Clinical Global Impression scale, *T1* after the 5-day in-person rehabilitation program, *T2* follow-up, *N* number of patients, improved category includes very much, much, and minimally improved; no change/worse category includes no change, minimally, much, and very much worse

*Statistically significant

## Discussion

Our results provide evidence that a 5-days in-person rehabilitation program followed by a 12-week telemedicine program can improve motor outcomes compared to home-based self-management, as measured by the decrease in S-FMDRS at the 3-months follow-up assessment. Improvement in motor symptom severity after the 5-days rehabilitation program was noted in both groups but it became more evident at the 3-months follow-up only in the telemedicine group. In contrast, the control group, which continued rehabilitation via a home-based self-management plan with no tele-sessions [30], scored significantly lower on motor symptom measures at the 3-months follow-up with performance comparable to T0. There was a significant improvement in physical fatigue (MFI-20 subscales) at 3-months follow-up as revealed by a significantly lower level of fatigue perceived by the telemedicine program group compared to the home-based self-management group. These results are in keeping with the fact that most telemedicine patients (94%) reported better self-perception of improvement at follow-up as revealed by the CGI. Some control group patients (18%) reported worsening of symptoms, while most reported an improvement (72%). Although fluctuation in symptoms might not be related to the rehabilitation plan overall, motor outcomes and physical fatigue in the medium term (3 months) seem to have stabilized in the telemedicine group.

The results for motor symptom severity (S-FMDRS) expand our recent findings: they are partially in keeping with the underlying improvement in S-FMDRS after the 5-days rehabilitation program but not at the 3-months follow-up assessment of the home-based self-management group [24]. Lower, albeit not significantly, S-FMDRS scores were recorded at the 3-months follow-up, but they were still significantly different from T0 [24], while in this study here reported the scores for home-based self-management group patients returned to baseline at T0. A possible explanation for this discrepancy could be that in our previous study we included the Exercise Adherence Rating Scale (EARS) [45] as a means to monitor adherence to the home-based exercises program. The lack of monitoring in the control group may have led to a significant worsening of motor disturbance scores at the 3-months follow-up due to a lower/discontinued adherence to the home-based self-management or a lack of exercises graded according to patient progress and condition.

The improvement in physical fatigue recorded for the telemedicine group is of notable importance, given that fatigue is a highly disabling non-motor symptom associated with FMDs [7, 12, 15]. Fatigue, more than the self-rated severity of motor symptoms, is the primary NMS that diminishes quality of life in these patients [15] and it underlines how tailored programs can have beneficial effects. While both groups showed similar improvement after completing the 5-days in-person rehabilitation program, physical fatigue was significantly lower at the 3-months follow-up in the telemedicine group. This is in line with our previous study and suggests that the physical fatigue component may respond to intensive rehabilitation treatments [24] and it confutes the idea that this disabling symptom hampers rehabilitation [30]. Also, the telemedicine program might have the advantage in the medium-term management that exercises can be graded according to a patient’s progress and condition.

The control group reported lower self-rated perception of physical fatigue at the 3-months follow-up; however, worsening of motor symptoms corroborates the finding that fatigue might be independent of motor symptom severity in patients with FMDs [15]. Differently for the telemedicine group, improvement in motor symptom severity was consistent with the decrease in physical fatigue. This leaves us wondering whether fatigue might be a core feature and part of the phenotype of FMDs [15]. If we assume that a critical element shared between fatigue and FMDs pathophysiology is the higher than usual effort needed to accomplish a task, we may then hypothesize that a mismatch between expectation and sensory feedback impacts this altered perception of effort (sensory attenuation) [46, 47]. We may further hypothesize that a decrease in effort might underlie improvement in both motor and physical fatigue, since the telemedicine program, but not the home-based self-management, provided for graded self-management. This underscores the importance of extending patient monitoring beyond the in-person rehabilitation phase by experts in the medium- and long-term without impacting health-care costs.

Moreover, at the 3-months follow-up, there was higher self-rated change perception (CGI) in the telemedicine group, but no significant differences in quality of life (SF-12). FMDs patients are noted feel misunderstood and abandoned by health-care professionals. Such dissatisfaction highlights inadequacies in the current clinical management of patients with FMDs, including inappropriate treatment (as well as rehabilitation) and powerlessness [33]. Designed by experts, the telemedicine program may have mitigated these feelings by extending appropriate treatment to the home and providing resources to empower patients in the management of their symptoms [33]. The reason why these effects did not parallel significant improvement in quality of life is not straightforward.

There is some inconsistency between the S-FMDRS score, which a clinician objectively rates during clinical examination, and the level of physical functioning as perceived by the control group but not the telemedicine group patients. The control group reported improvement in CGI scores at follow-up despite worsening of motor symptoms: there was a mismatch between the patients' perception of the severity of their motor symptoms (CGI) and the severity revealed by more objective measures such as clinical examination (S-FMDRS) [48, 49]. The difference implies the primary efficacy of the 5-days rehabilitation program, despite lack of motor symptom stabilization. Another factor may be the difference in sensory perception, in agreement with the Bayesian model [47], resulting in a general altered awareness.

Finally, regarding the other motor aspects (gait and posture), fatigue (general, reduced activity, reduced motivation, mental), and pain, the present results are consistent with our preliminary data [24], suggesting a predominant role of the disease-specific management of these patients regardless of the telemedicine program. We noted a general improvement over time in gait speed, cadence, stride length, and postural control (decrease in CoP length), in the general aspects of fatigue related to activity and motivation, and in the mental domain of QoL, regardless of the type of treatment. Similarly, for the non-motor outcomes measured only at T0 and T2, we found an effect of time in both groups, underlining the primary effect of the 5-days rehabilitation program. Improvement in scores on the BDI-II, BAI, TAS-20, and SF-12 physical functioning was observed in both groups. The significant effects of Group on depressive symptoms (BDI–II) and mental health of QoL (SF-12) revealed overall better improvement in the telemedicine group. This suggests an increase in the mental aspect of QoL and mood and a reduction in powerlessness perceived by the telemedicine group. Psychotherapy treatment given remotely once a week for 12 weeks in psychogenic nonepileptic seizures has proven beneficial [50]. In the future, physical rehabilitation combined with psychological support could lead to better improvement in several outcome measures and especially non-motor measures, given that a multidisciplinary approach could be best suitable, in line with the biopsychosocial model that frames functional neurological disorders [51].

As in our previous study we found no effect on pain, which suggests a lack of pain management interventions. Adamse and colleagues [52] reported no effect on pain in their review regarding telemedicine and usual care in patients with chronic pain. The lack of worsening of pain suggests that pain does not contraindicate rehabilitation. Future telemedicine trials with longer-term programs that include pain self-management (i.e., cognitive-behavior therapy) might shed more light on how to better manage this disabling symptom.

We believe that our preliminary data hold twofold significance. From a clinical perspective, the data show for the first time the importance of integrating an evidence-based in-person rehabilitation program with a telemedicine program to extend disease-specific management by experts in the medium term. Adjuncts and innovations to improve access to specialist rehabilitation treatment by qualified professionals aided by advanced devices (i.e., tele/remote health and wearable technology) and long-term monitoring have seldom been explored in patients with FMDs [19, 34]. By exploring a staged approach to FMDs, such as acute admission to specialized centers followed by outpatient programs ensuring appropriate treatment, the burden on the care system can be reduced [27, 28]. Within this perspective, telemedicine offers many advantages: (1) regular communication between clinicians and real-time assessment of the patient’s environment; (2) patient improvement in satisfaction and quality of life, cutting the cost and labor of accessing health care; (3) improved quality of services by monitoring patients in their home rather than traveling to distant treatment centers can also improve the cost-effectiveness of interventions [53]. The recent scoping review by Gilmour and Jenkins [28] reported that while rehabilitation was consistently used as an inpatient intervention with positive results, most patients returned to independent function at discharge despite persistence of symptoms.

Telemedicine offers the potential to extend specific paths from the hospital to the home phase and provides for better management of disease by highly qualified personnel and for enhancement of clinical, social, and economic outcomes. This is crucial in treating FMDs because of the clinical complexity of patients who require highly qualified personnel, adaptation of rehabilitation programs according to patient progress, and long-term monitoring without impacting health-care costs.

The COVID-19 pandemic has prevented patients from accessing rehabilitation in hospital settings. The lack of a sufficient number of specialist centers for the rehabilitation of patients with FMDs emphasizes the need to create telemedicine pathways with qualified staff to reach patients unable to access rehabilitative treatments. Given that the protocol was carried out during the pandemic, someone might argue that such event could have altered the experience of presenting FMDs, and their related outcomes. Indeed, an increased incidence of FMDs have been reported [54], showing that the pandemic might represent a risk factor for developing the disorder. Nevertheless, two surveys on our cohort of FMD patients revealed a general stability over time of both motor and non-motor symptoms, revealing that patients undergoing multidisciplinary management did not display an increased vulnerability [55, 56]. NMSs, including pain, however, need to be routinely assessed, and attention paid to the patients during treatment. From a scientific perspective, our data provide preliminary reference for the design of randomized controlled trials on the effects of telemedicine programs in the management of patients with FMDs.

Our study has several limitations: first, the study design, conceived to provide preliminary data for conducting an RCT study, might have influenced the differences found in the Telemedicine group compared to the self-management group at T1 (except for the CGI, scores are always better in the Telemedicine group) due to overall improvements in the treatment service; second, the presence of more patients with gait impairments in the telemedicine group might have been balanced by more isolated FMDs in the control group on our outcomes, as both phenotypes might show better improvement (but studies avoiding such differences should be preferred). Third, the lack of sample size estimation, the open-label design, the lack of blinded raters in S-FMDRS assessment and the absence of long-term follow-up at the end of the 12-week program (home-based/telemedicine). Moreover, some measures were collected only at T0 and T2, precluding the recognition of the relative contribution of rehabilitation or the self-management/telemedicine program; no specific exercises were included to target NMSs independently. Finally, our explorative analysis allows only for preliminary exploration of the data collected so far: we did not measure whether patients left work because of the disorder and were subsequently able to return to it after the rehabilitation program, and we did not include data on the economic impact of our program, a potential important outcome to take into account for future studies [8].

The strengths of our study are the use of: a validated 5-days rehabilitation protocol; a comprehensive assessment battery to capture motor and NMSs, including objective-rated and patient-rated outcome measures [17]; instrumental gait and balance assessment to obtain quantitative data of performance.

## Conclusions

Telemedicine is at the frontier of managing neurological disabilities [57, 58], including FMDs. Our data support the combination of two innovative approaches to FMDs (5-days physiotherapy and telemedicine) and a shift in attention toward overcoming geographical barriers to access specialist treatment. The implementation of telemedicine programs could help stabilize symptom fluctuation and reduce the need for further care provided by the national health service and the costs that such disorders incur, and ultimately improve the quality of life of patients and caregivers [8]. A fundamental step in this direction was to explore the efficacy of a simple telemedicine program to be implemented in future RCTs including outcomes on employment and economic costs for the health service [8].

## Data Availability

The data sets analyzed during the current study are available upon request with no restrictions.
